# Preparation of a novel antiserum to aromatase with high affinity and specificity: Its clinicopathological significance on breast cancer tissue

**DOI:** 10.1371/journal.pone.0177439

**Published:** 2017-05-10

**Authors:** Naoki Kanomata, Shiro Matsuura, Tsunehisa Nomura, Junichi Kurebayashi, Taisuke Mori, Jo Kitawaki, Takuya Moriya

**Affiliations:** 1 Department of Pathology, Kawasaki Medical School, Kurashiki, Japan; 2 Corporate Planning Department, LSI Medience Corporation, Tokyo, Japan; 3 Department of Breast and Thyroid Surgery, Kawasaki Medical School, Kurashiki, Japan; 4 Department of Obstetrics and Gynecology, Kyoto Prefectural University of Medicine, Graduate School of Medical Science, Kyoto, Japan; State University of New York Upstate Medical University, UNITED STATES

## Abstract

Aromatase inhibitors have been widely used for the endocrine treatment of estrogen-dependent breast cancer in postmenopausal patients. However, clinicopathological studies of aromatase have been limited due to unsatisfactory specificity and/or restricted availability of anti-aromatase antibodies. Here, we have generated a polyclonal antiserum with high affinity and specificity for human aromatase using a monoclonal antibody tagged immunoaffinity chromatography on an industrial production scale. Our preliminary immunohistochemical analysis of 221 invasive breast cancer cases indicated that 87.3% (193/221) had at least 5% aromatase positive cells. The histoscore for aromatase was inversely correlated with pT (*p* = 0.019), pN (*p* = 0.001), stage (*p* < 0.001), histologic grade (*p* = 0.003), lymphatic infiltration (*p* < 0.001), venous infiltration (*p* < 0.001), and Ki-67 index (*p* < 0.001). However, cancer aromatase expression was independent of estrogen receptor (ER), progesterone receptor (PgR), and human epidermal growth factor receptor 2 statuses. This antiserum will be applicable to clinicopathological examination of aromatase in addition to ER and PgR for an appropriate use of aromatase inhibitor on the treatment of breast cancer. Further studies on the relationship between Aromatase inhibitors have been widely used for the endocrine treatment of estrogen-dependent breast cancer in postmenopausal patients. However, clinicopathological studies of aromatase have been limited due to unsatisfactory specificity and/or restricted availability of anti-aromatase antibodies. Here, we have generated a polyclonal antiserum with high affinity and specificity for human aromatase using a monoclonal antibody tagged immunoaffinity chromatography on an industrial production scale. Our preliminary immunohistochemical analysis of 221 invasive breast cancer cases indicated that 87.3% (193/221) had at least 5% aromatase positive cells. The histoscore for aromatase was inversely correlated with pT (*p* = 0.019), pN (*p* = 0.001), stage (*p* < 0.001), histologic grade (*p* = 0.003), lymphatic infiltration (*p* < 0.001), venous infiltration (*p* < 0.001), and Ki-67 index (*p* < 0.001). However, cancer aromatase expression was independent of estrogen receptor (ER), progesterone receptor (PgR), and human epidermal growth factor receptor 2 statuses. This antiserum will be applicable to clinicopathological examination of aromatase in addition to ER and PgR for an appropriate use of aromatase inhibitor on the treatment of breast cancer. Further studies on the relationship between aromatase expression and aromatase inhibitors are warranted.

## Introduction

Breast cancer is one of the most common cancers in the world, affecting women with a prevalence of more than 10% in the general population [1]. The estrogen-activated estrogen receptor is a key driver of the breast cancer phenotype in around 70% of patients [2, 3]. Tamoxifen, one of the antagonists of estrogen receptor (ER) in mammary tissues, is a well-established and effective treatment for both premenopausal and postmenopausal patients with ER-positive breast cancers. Furthermore, tamoxifen significantly reduces the risk of estrogen-dependent breast cancer [3]. On the other hand, inhibition of aromatase activity is now a key approach in treating estrogen-dependent breast cancers, because aromatization of androgens is the last and rate-limiting step in estrogen synthesis. Aromatase is expressed at higher levels in breast cancer tissues than in normal mammary tissues [4–6]. *In situ* produced estrogen from overexpressed aromatase in breast cancer cells is thought to play a crucial role in stimulating cancer cell growth. Third-generation aromatase inhibitors, anastrozole, letrozole, and exemestane, have been widely used for the endocrine treatment of estrogen-dependent breast cancer in postmenopausal patients [7, 8].

To develop individual therapies for patients with breast cancers, the expression levels of ER, progesterone receptor (PgR), and human epidermal growth factor receptor 2 (HER2) in tumor specimens are currently evaluated using immunohistochemistry. Once ER and/or PgR are detected, the specimens are considered estrogen dependent and endocrine therapies including ER antagonists and aromatase inhibitors are introduced for patients with early stage cancers. However, in theory the response to aromatase inhibitors is not regulated simply by hormone receptor status.

The accurate evaluation of aromatase expression and activity to predict the efficacy of aromatase inhibitors in treating patients with breast cancer has been extremely difficult to use clinically. Indeed, although a number of antibodies to aromatase expression have been developed, they do not always show satisfactory results, especially when used for immunohistochemistry. Moreover, these are limited to laboratory use and are not applicable to large-scale clinical examinations [9–15]. A monoclonal antibody 677 has produced valuable information for aromatase. Unfortunately, this is not available commercially [16, 17]. Here, we produced polyclonal antisera to MAb3-2C2 immunopurified human placental aromatase on an industrial production scale. To test whether the antiserum would be applicable to clinicopathological examination of aromatase in breast cancer tissues, we conducted a preliminary immunohistochemical analyses in the tissue microarrays constructed with our invasive breast cancer cases. We studied the association of aromatase status with various clinicopathological factors.

## Materials and methods

### Immunopurification of human placental aromatase

Hybridoma cells producing a monoclonal antibody specific to human placental aromatase (MAb3-2C2)[10, 13] were subcloned and inoculated intraperitoneally to pristane-primed adult female BALB/C mice. After 10–14 days, ascites fluids were collected. Immunoglobulin G (IgG) was purified by rProtein A Sepharose Fast Flow chromatography (GE Healthcare, Chicago, IL, USA) according to the manufacturer’s instructions and stored at –40°C. The purity of IgG was examined with sodium dodecyl sulfate–polyacrylamide gel electrophoresis (SDS–PAGE) as described below and was shown to be pure. An immunoadsorbent substrate was prepared by coupling MAb3-2C2 (9 mg of IgG/mL of matrix) with CNBr-activated Sepharose 4B (GE Healthcare) as described [11].

Microsome pellets of human term placentas were prepared as described [11] and were stored at −40°C. Aromatase was purified from microsome pellets according to Yoshida and Osawa [11] with some modifications. Briefly, the microsome pellets (approximately 45 mL) were suspended in an ice-water bath by adding 90 mL of 10 mM phosphate buffer (PB; pH 7.4) containing 0.1 mM EDTA, 5.0 μM androstenedione (Tokyo Kasei, Tokyo, Japan), and 20% glycerol. To this stirred suspension we added 10% Emulgen 1108 (Kao Chemicals, Tokyo, Japan) and 10% sodium cholate solutions to a final concentration of 0.15% each. The mixture was stirred for 30 min at 4°C and centrifuged at 250,000 *g* for 60 min. The supernatant fluid (approximately 100 mL) was passed through a Sepharose 4B column coupled with MAb3-2C2 (9 mL of column bed volume) at a flow rate of 7 mL/h at 4°C. The column was then washed with 45 mL of 10 mM PB containing 0.1 mM EDTA, 5.0 μM androstenedione, 20% glycerol, and 0.15% Emulgen 1108, and was washed additionally with 135 mL of 10 mM PB containing 0.1 mM EDTA, 5.0 μM androstenedione, 20% glycerol, 0.15% Emulgen 1108, and 0.5 M NaCl. Then, the immunopurified aromatase was eluted with 90 mL of 10 mM PB containing 0.1 mM EDTA, 5.0 μM androstenedione, 20% glycerol, 0.15% Emulgen 1108, and 4.0 M NaCl. The eluate was concentrated by an ultrafiltration with a Vivaspin20^®^ centrifugal concentrator (molecular weight cutoff 10,000; VS2002; Sartorius, Göttingen, Germany) at 4°C at 300 *g* to a final volume of 1.5 mL. Then, the concentrated aromatase fraction was resuspended with 20 mL of 10 mM phosphate-buffered saline (PBS; pH 7.4) containing 0.1 mM EDTA, 5.0 μM androstenedione, 20% glycerol, 0.15% Emulgen 1108, 0.01% SDS, and was concentrated to a final volume of 1.0 mL by an additional ultrafiltration step. The protein concentration of purified aromatase was determined using BCA Protein Assay kits (Pierce Biotechnology, Rockford, IL) with bovine serum albumin (Pierce Biotechnology) as the standard. The purified aromatase was stored at −40°C in siliconized glassware.

### Preparation of rabbit polyclonal aromatase antisera

Two female New Zealand White rabbits (Rabbits 1 and 2) were immunized according to Kitawaki et al. [13], namely 200 μg of purified aromatase solution was injected intracutaneously in the back after mixing with an equal volume (165 μL) of Freund’s complete adjuvant. At 12 weeks after the first immunization, the rabbits were given booster injections of 70 μg of the same antigen mixed with an equal volume (165 μL) of Freund’s incomplete adjuvant. Another rabbit (Rabbit 3) was immunized intracutaneously in the back with 100 μg of purified aromatase with an equal volume (83 μL) of Freund’s complete adjuvant. Two weeks after the first immunization, this rabbit was given a booster injection of 100 μg of the same antigen mixed with an equal volume (83 μL) of Freund’s incomplete adjuvant. At 2 and 8 weeks after the booster injection, the same rabbit was given intravenous injections twice with 100 μg aliquots of the same antigen. One week after the final immunization, blood was drawn from each of the three rabbits and stored at −40°C.

### SDS–PAGE and amino acid sequencing

SDS–PAGE was performed using pre-cast NuPAGE 4–12% Bis-Tris gels (Invitrogen, Carlsbad, CA, USA) with a 1:20 dilution of MOPS-SDS running buffer (Invitrogen) according to the manufacturer’s instructions. The protein bands were stained with 0.05% Coomassie Brilliant Blue (CBB). The purity of aromatase was determined by scanning each protein band with a CS-9000 densitometer (Shimadzu, Kyoto, Japan) after separation with SDS–PAGE and staining with CBB.

N-terminal amino acid sequencing of purified aromatase was performed using a PPSQ-23A protein sequencer (Shimadzu) at Toray Research Center (Tokyo, Japan) after electrotransferring the protein bands separated by SDS–PAGE to polyvinylidenedifluoride (PVDF) membranes (Immobilon PSQ, Millipore, Temecula, CA, USA) and staining them with 0.1% Amide Black.

### Western blotting

The PVDF membranes were cut into 10 × 10 cm squares and preconditioned with 20% methanol. Proteins separated by SDS–PAGE were electrotransferred to a membrane at 15 V for 2 h at room temperature. After the remaining binding sites to protein were blocked with 50 mM PBS containing 10% skim milk (Wako Pure Chemicals, Osaka, Japan), the membrane was incubated with a 1:1000 dilution of rabbit antisera in PBS containing 1% skim milk and 0.1% Tween 20 (Bio-Rad, Hercules, CA, USA) for 2 h at room temperature. Anti-aromatase antiserum R-8-1 specific for human placental aromatase [13] was used as the positive control. The membranes were washed with PBS containing 0.1% Tween 20 and was incubated with a 1:1000 dilution of horseradish peroxidase (HRP)-conjugated anti-rabbit Ig (DAKO, Glostrup, Denmark) in PBS containing 1% skim milk and 0.1% Tween 20 for 1 h at room temperature. The membranes were washed with PBS containing 0.1% Tween 20, and then incubated with HRP development reagent (Bio-Rad) according to the manufacturer’s instructions to develop protein bands bound to the HRP conjugate.

### Enzyme-linked immunosorbent assay (ELISA)

Purified aromatase, a human placental microsome extract, and a liver microsome extract from Mixed Gender Pooled Human Liver S9 (Bioreclamation IVT; Hicksville, NY; 21 mg protein/mL) were diluted with PBS to final concentrations of 5, 50, and 500 μg/mL, respectively, as immobilizing antigens. A 50-μL aliquot of each antigen solution was dispensed to each well of 96-well microplates (Immulon 2HB, Thermo Scientific, Waltham, MA). The plate was incubated at 4°C overnight and the wells were washed with PBS. A 300 μL aliquot of a 1:10 dilution of SuperBlock (SkyTec Lab, West Logan, UT) in PBS containing 0.1% Tween 20 and 1 mM EDTA was dispensed into each well. The plate was incubated at 25°C for 1 h to block the remaining binding sites to protein, and the wells were washed with PBS containing 0.1% Tween 20. A 50 μL aliquot of 2-fold serial dilutions (ranging from 1:1,000 to 1:16,000) of each antiserum in a 1:10 dilution of SuperBlock in PBS containing 0.1% Tween 20 and 1 mM EDTA was dispensed to each well. The plate was incubated at 25°C for 1 h and the wells were washed with PBS containing 0.1% Tween 20. A 50 μL aliquot of a 1:1,000 dilution of HRP conjugated anti-rabbit IgG in a 1:10 dilution of SuperBlock in PBS containing 0.1% Tween 20 and 1 mM EDTA was dispensed to each well. The plate was incubated at 25°C for 1 h and the wells were then washed with PBS containing 0.1% Tween 20. A 50 μL aliquot of 4.2 m M3, 3′, 5, 5′-tetramethylbenzidine (Wako Pure Chemicals) solution in 100 mM acetate buffer (pH 5.5) containing 0.006% H_2_O_2_ and 1 mM EDTA was dispensed to each well and incubated at room temperature to start the enzyme reaction. After 5 min for purified aromatase-immobilized wells and 15 min for placental and liver microsome-immobilized wells, the enzyme reaction was stopped by dispensing a 50 μL aliquot of 1 M HCl to each well. The light absorbance of each well was measured at 450 nm wavelength using an EL312e96-well microplate reader (BIO-TEK Instruments, Winooski, VT).

### Immunohistochemistry

A consecutive series of surgically resected invasive breast cancers was used for this study. A total of 236 invasive breast cancers were resected at the Department of Breast and Thyroid Surgery, Kawasaki Medical School, Kurashiki, Japan, from June 2009 to December 2010. The local ethics committee granted approval for this study (Institutional Review Board of Kawasaki Medical School, approval number 1783). Written informed consent was received from all patients. Five microinvasive carcinomas were excluded from this study, because of the lack of sufficient material. Clinical information was collected from the hospital charts. Human term placenta and normal liver tissues were used for positive and negative controls, respectively. Representative paraffin blocks were extracted and tissue microarrays were constructed using a KIN-2 system (Azumaya Inc., Tokyo, Japan) that punches out tissues using a 2-mm needle. Immunostaining was performed using the EnVision Plus kit (Dako). Thin 4-μm sections were cut from the tissue microarrays. After dewaxing and hydration, they were placed in a hot bath of Target Retrieval Solution, pH 9.0 (Dako), at 95°C for 40 min for ER, PgR, and Ki-67. The sections were incubated with the primary antibodies for 30 min (ER, PgR, and Ki-67) or 1 h (aromatase) at room temperature. The primary antibodies were: anti-aromatase antiserum from Rabbit 3 at a dilution of 1:1,000; an anti-ER monoclonal antibody (clone: 1D5, Dako) at a dilution of 1:50, an anti-PgR monoclonal antibody (clone: PgR636, Dako) at a dilution of 1:800; and an anti-Ki-67 monoclonal antibody (clone: MIB-1, Dako) at a dilution of 1:50. The chromogen used was 3,3′-diaminobenzidine tetrachloride, and the sections were counterstained with hematoxylin. HER2 expression was tested using a HercepTest (Dako) according to the manufacturer’s instructions. For double immunohistochemistry, first immunohistochemical staining of ER, PgR or Her2 was performed as usual method, and the slides were then incubated with anti-aromatase antiserum followed by EnVision-AP (Dako) and Vulcan Fast Red (Biocare Medical, Concord, CA). Immunohistochemistry for aromatase was analyzed using a histoscore that was calculated by multiplying the positive area (%) and intensity (0–2: 0 for negative, 1 for weak, 2 for strong staining). The evaluation of the immunohistochemistry staining was manually double-checked by a pathologist (N. K.) in a blinded manner and cross-checked by a senior pathologist (T. M.). Aromatase expression was evaluated only in cancerous epithelium and expression in the stromal tissues was not counted. The pT, pN and stage were determined according to the 7th edition of the UICC TNM Classification [18].

### Statistical analyses

Statistical analyses were performed using IBM SPSS Statistics (version 23; IBM Corp., Armonk, NY). The χ2 test was used for analyzing pN factor, lymphatic infiltration, ER, PgR and HER2; Fisher’s exact test was used for pT factor, distant metastasis, stage and venous infiltration; and the Mann–Whitney nonparametric *U* test was used for age, histological grade and Ki-67 index to identify significant differences in the frequencies of the clinicopathological factors and aromatase immunohistochemical results. *P* < 0.05 was considered significant.

## Results

### Purification of aromatase

**Fig 1:**
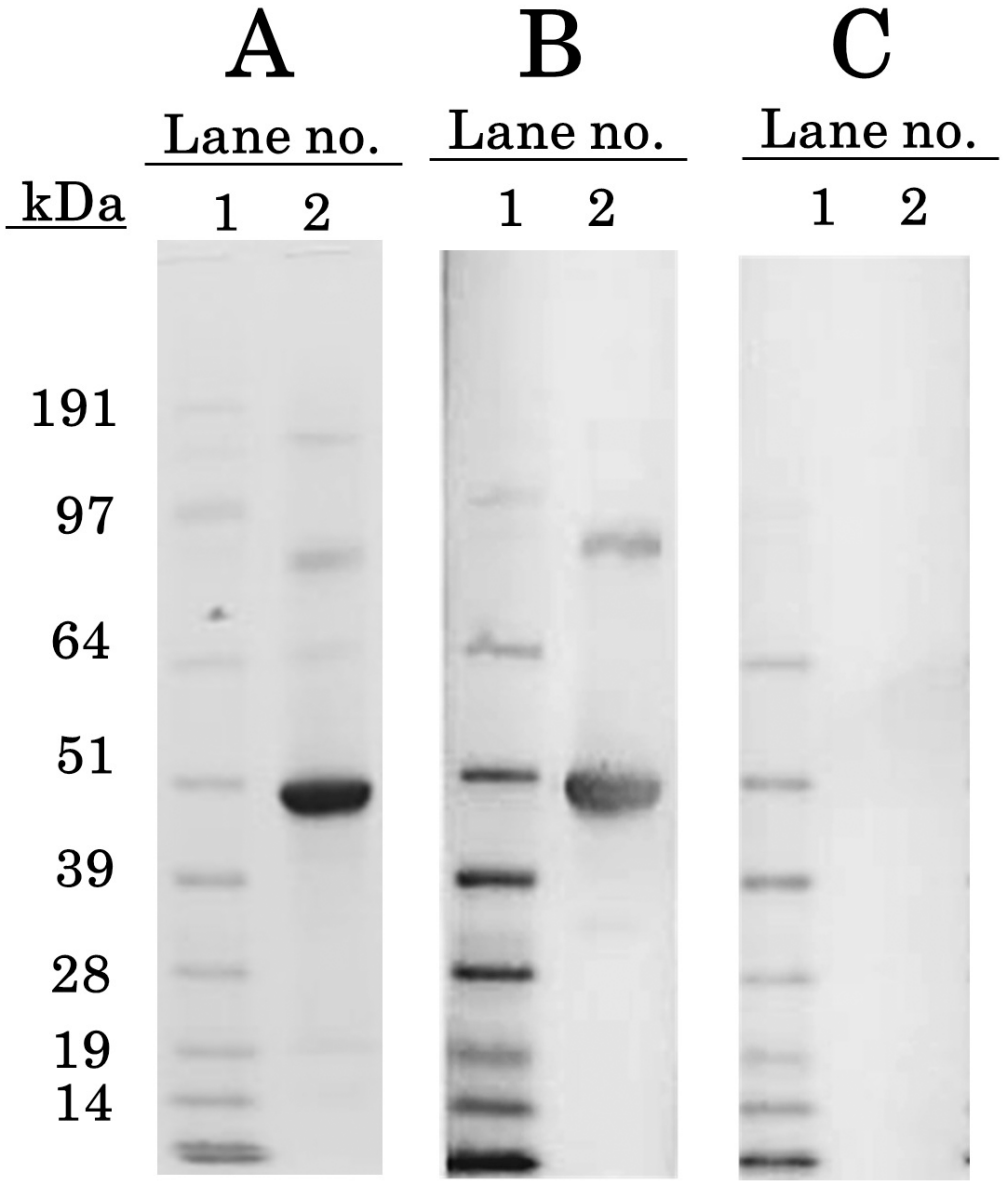
SDS–PAGE separation and western blotting of purified aromatase. Microsome pellets of human term placenta were solubilized and applied to an immunoadsorbent column coupled with a monoclonal antibody (MAb3-2C2) specific to aromatase. The eluted aromatase was subjected to SDS–PAGE and western blotting. A, SDS–PAGE separated fractions of the purified aromatase (stained with coomassie brilliant blue: CBB). B, western blot of aromatase (immunostained with R-8-1, a polyclonal antiserum specific to aromatase). C, negative control (reacted with normal rabbit serum). Lanes: 1, molecular weight markers; 2, the purified aromatase.

We used immunoadsorbent columns coupled with MAb3-2C2 that had been established as specific for human placental aromatase, and purified a total of 1.218 mg aromatase from approximately 45 mL human placental microsomal pellets. SDS–PAGE was used to detect a main band flanking to c. 50 kDa, and three minor bands flanking to c. 58 kDa, c. 95 kDa, and c. 190 kDa (Fig 1A). Western blot analyses revealed that all four bands, but not others, immunostained with the anti-aromatase antiserum R-8-1 (Fig 1B). Of these bands, the two flanking to c. 58 kDa and c. 190 kDa were broad and their color intensities were very weak. As a result, these two bands could not be presented adequately in Fig 1B. N-terminal amino acid sequencing of the main band flanking to c. 50 kDa gave two patterns of amino acid sequences: Val·Leu·Glu·Met·Leu and Met·Leu·Asn·Pro·Ile. These sequences were identical to amino acids 2–6 and 5–9 from the N-terminal of aromatase, respectively, indicating that the main band was a mixture of the n-1 and n-4 forms of the aromatase monomer [19] (S1 Fig). The other minor bands were estimated to be different molecular sizes of monomers and agglutinations. As the sum of the three bands—50, 95 and 190 kDa—was calculated to cover 92% of the total protein, we omitted further purification.

### Rabbit polyclonal antisera to aromatase

**Fig 2:**
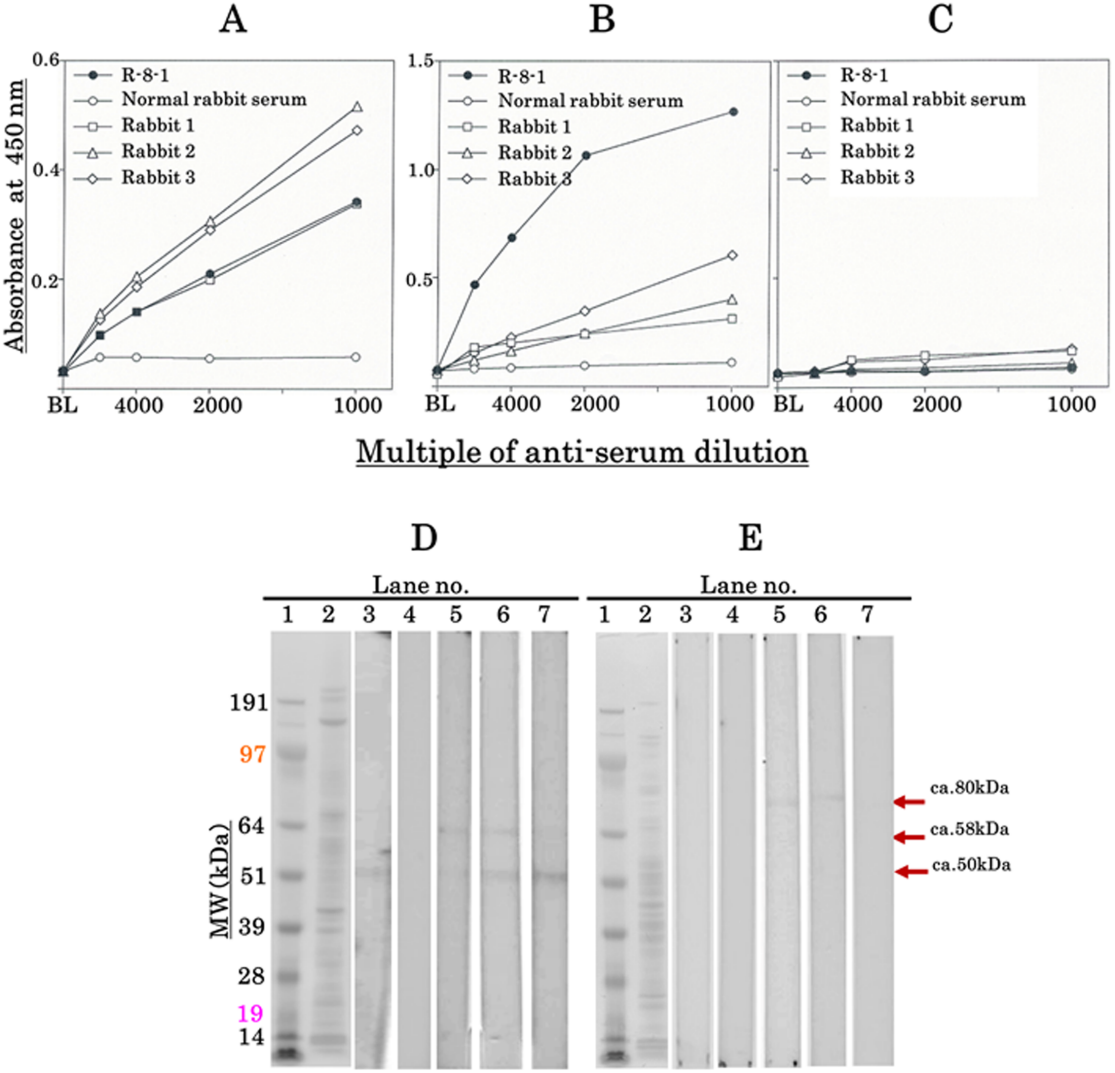
Reactivity of antisera to aromatase. The sensitivity and specificity of antisera to aromatase were evaluated by an ELISA using A, purified aromatase; B, a placental microsome extract; and C, a liver microsome extract, as immobilized antigens. BL, blank. Western blotting with antisera to aromatase for D, a placental microsome extract, and E, a liver microsome extract. The extracts were separated by SDS–PAGE and electrotransferred to a PVDF membrane. This was subjected to western blotting with each of three antisera (Rabbits 1, 2, and 3) using antiserum R-8-1[11–13] as the positive control. Lanes: 1, molecular weight markers; 2, CBB staining; 3, R-8-1; 4, normal rabbit serum (negative control); 5, Rabbit 1; 6, Rabbit 2; and 7, Rabbit 3.

We immunized three rabbits with purified aromatase and obtained approximately 30 mL of antisera from each of them. Each antiserum was subjected to antigen-immobilized ELISA at dilutions ranging from 1:1,000 to 1:16,000 to examine the sensitivity and specificity to purified aromatase, placental microsomal extract, and liver microsomal extract (Fig 2A–2C). Each of the antisera revealed equal or a 1.5-fold better affinity to the purified aromatase compared with R-8-1. However, each of the three antisera revealed considerably lower affinity to placental microsomal extract than R-8-1. The three antisera and R-8-1 all showed very little nonspecific affinity to liver microsomal extracts.

Western blotting revealed that each of the three rabbit antisera detected the c. 50 kDa band (consistent with an aromatase monomer) and the c. 58 kDa band of placenta microsomal extract (Fig 2D and 2E). However, the antisera from Rabbits 1 and 2 detected a faint nonspecific c. 80 kDa band from the liver microsomal extract. The 95 kDa band was specific for aromatase; the 80 kDa band was seen in the reaction of Rabbit 1 and 2, but not in R-8-1 or the normal rabbit control. We concluded that the 80 kDa band represented a nonspecific reaction for Rabbit 1 and 2, as no 80 kDa band was seen in the assay for Rabbit 3. Thus, the antiserum from Rabbit 3 proved the most specific against aromatase among the three tested.

**Fig 3:**
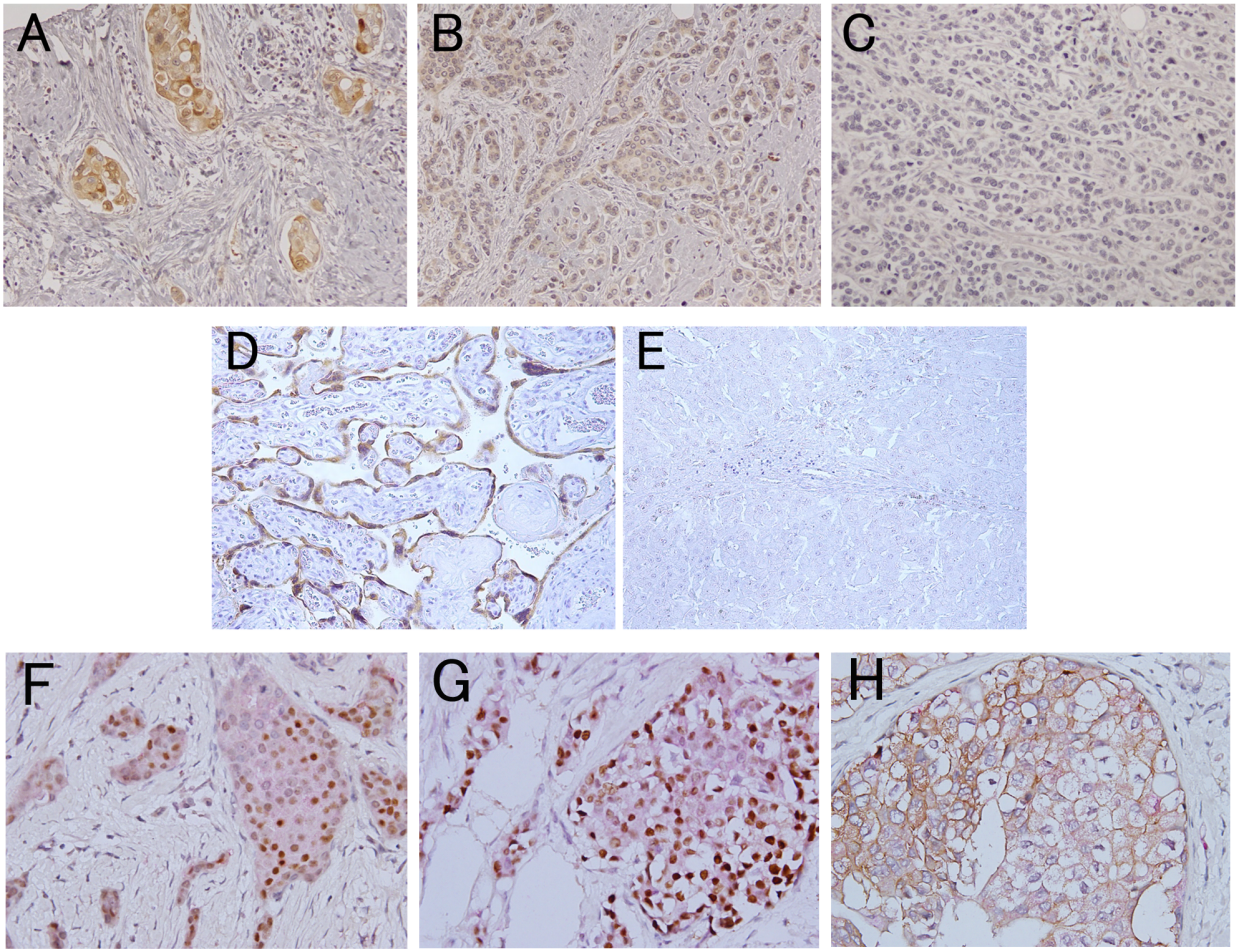
Immunohistochemistry of aromatase in breast cancer tissues. A, strong aromatase expression of a case that was negative for ER, PgR and HER2, with histologic grade 2 and pT1bN0M0. B, weak aromatase expression of a case that was positive for ER and PgR; negative for HER2, with histologic grade 2 and pT1cN1aM0. C, negative aromatase expression of a case that was positive for ER and PgR; negative for HER2, with histologic grade 2 and pT1cN0M0. D, positive control from human term placenta. E, negative control from normal human liver. F, double immunostaining for ER (brown) and aromatase (red) in tissue that was positive for ER, PgR, and negative for HER2, with histologic grade 2 and pT1cN0M0. G, double immunostaining for PgR (brown) and aromatase (red) in tissue that was positive for ER, PgR and negative for HER2, with histologic grade 2 and pT1cN0M0. H, double immunostaining for HER2 (brown) and aromatase (red) in tissue that was negative for ER, PgR and positive for HER2, with histologic grade 2 and pT1cN3aM0.

Immunohistochemistry using antiserum from Rabbit 3 revealed intense and specific staining in the cytoplasm of syncytiotrophoblasts of human term placenta, compared with completely negative staining in normal human liver specimens (Fig 3D and 3E). This demonstrated that the antiserum was highly specific to aromatase with little cross-reactivity to other antigens including other cytochrome p450 enzymes. Aromatase localization in the placenta was consistent with previous findings using the polyclonal antiserum R-8-2 [20]. In breast tissues, aromatase was expressed in normal and neoplastic cells. Epithelial aromatase expression was consistently stronger than stromal aromatase expression (Fig 3A–3D).

### Clinicopathological analyses

The age of patients ranged from 32 to 93 years with a median of 59 and a mean of 58.1. All patients were female, and all of the tumors were unilateral. The other clinicopathological factors are shown in Table 1.

**Table 1:** Summary of clinicopathological factors.

	Cases	%		Cases	%
Surgery			pT factor		
total mastectomy	96	43.4%	pT1a	16	7.2%
breast-conserving surgery	125	56.6%	pT1b	50	22.6%
			pT1c	98	44.3%
Histology			pT2	50	22.6%
invasive carcinoma of no special type	194	87.8%	pT3	4	1.8%
invasive lobular carcinoma	13	5.9%	pT4b	3	1.4%
mucinous carcinoma	7	3.2%			
invasive micropapillary carcinoma	4	1.8%	pN factor		
apocrine carcinoma	2	0.9%	pN0	150	67.9%
metaplastic spindle cell carcinoma	1	0.5%	pN1	40	18.1%
			pN2	18	8.1%
Histologic grade			pN3	13	5.9%
G1	79	35.7%			
G2	99	44.8%	Distant metastasis		
G3	43	19.5%	absent	218	98.6%
			present	3	1.4%
Nuclear grade					
G1	132	59.7%	Pathological stage		
G2	43	19.5%	IA	129	58.4%
G3	46	20.8%	IB	1	0.5%
			IIA	40	18.1%
Lymphatic infiltration			IIB	18	8.1%
absent	110	49.8%	IIIA	18	8.1%
present	111	50.2%	IIIB	1	0.5%
			IIIC	11	5.0%
Venous infiltration			IV	3	1.4%
absent	182	82.4%			
present	39	17.6%	Primary systemic therapy		
			yes	12	5.4%
ER			no	209	94.6%
negative	35	15.8%			
positive	186	84.2%	Adjuvant hormonal therapy		
			yes	169	76.5%
PgR			no	52	23.5%
negative	67	30.3%			
positive	154	69.7%	Adjuvant chemotherapy		
			yes	97	43.9%
HER2			no	124	56.1%
negative	189	85.5%			
positive	29	13.1%			
equivocal	3	1.4%			

### Aromatase status in relation to clinicopathological factors

Of the tissue microarrays originally constructed with 231 cases, 10 did not have enough tissue for evaluation, so we finally assayed 221 cases for aromatase expression. One hundred ninety-three cases (87.3%) had at least 5% aromatase-positive cells. The histoscore for aromatase ranged from 0 to 200 with a median of 40 and mean of 52.9. The histoscore was significantly correlated with pT, pN, distant metastases, and tumor stages. The aromatase histoscore was higher in the pT1–2 cases than the pT3–4 cases (Mann–Whitney U; *p* = 0.019); higher in the cases without nodal metastasis than in those with nodal metastasis (*p* = 0.001); and higher in the stage I or II cases than in stage III or IV cases (*p* < 0.001). However, there were no significant correlations between the aromatase histoscore and distant metastasis, ER, PgR, or HER2 status (*p* = 0.114, *p* = 0.543, *p* = 0.313, and *p* = 0.280, respectively) (Fig 3F–3H).

**Fig 4:**
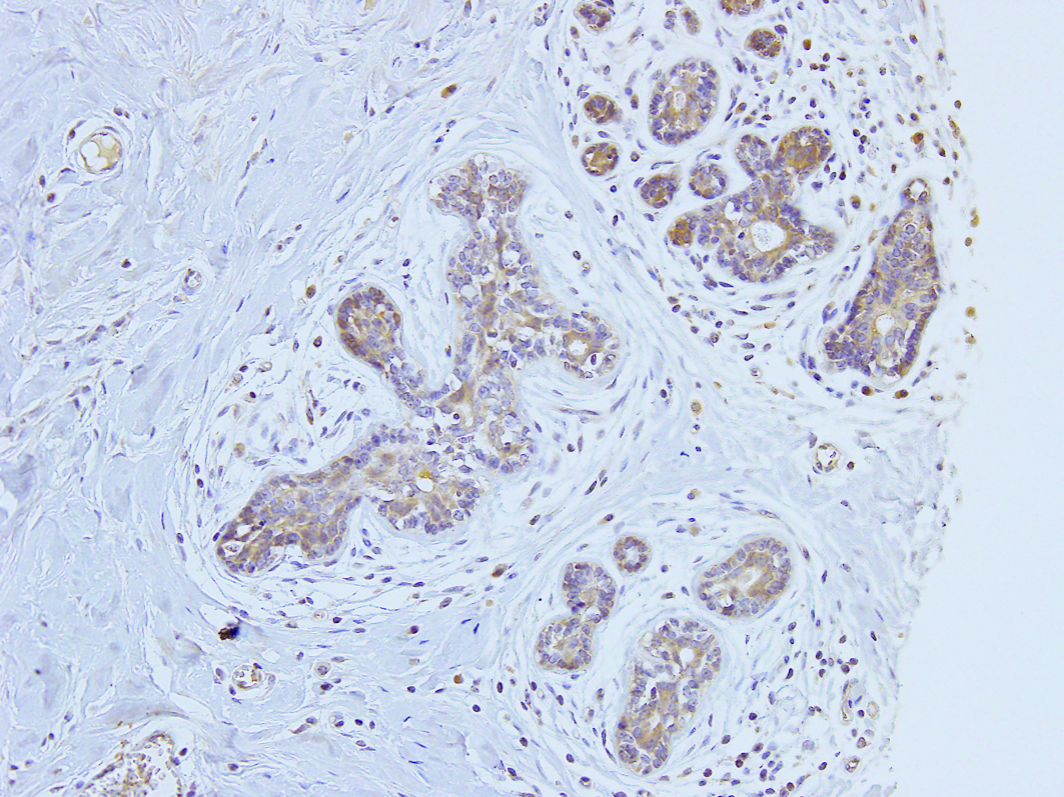
Immunohistochemistry of aromatase in normal breast tissue.

When the histoscores were divided into high and low groups by the median value (40), the patient’s age was significantly greater in the high group than in the low group (*p* = 0.012). The histological grade and Ki-67 labeling index were significantly higher in the low histoscore group than in the high group (*p* = 0.003 and *p* < 0.001, respectively; Table 2). The aromatase histoscore was significantly and inversely correlated with lymphatic infiltration (*p* < 0.001) and venous infiltration (*p* < 0.001; Table 2). Ninety-two of the 186 ER-positive cases showed high expression of aromatase (49.4%), while 18 cases among the 35 ER-negative cases showed high expression of aromatase (51.4%; *p* = 0.976). We also evaluated aromatase expression in normal breast epithelium adjacent to tumor cells in 46 cases. All the normal breast epithelium samples showed aromatase expression (46/46, 100%); the histoscore of these ranged from 30 to 200 with a median of 90 and mean of 106.3. Aromatase expression was seen in cytoplasm of normal breast epithelium (Fig 4).

**Table 2:** Correlation between aromatase expression level and various clinicopathological factors.

	Low aromatase	High aromatase	*P*
Age (y)	56 (56.0 ± 11.9)^a^	62 (60.1 ± 13.5)	0.012*
pT factor:			
pT1–2	104	110	0.065
pT3–4	6	1	
pN factor:			
pN0	68	83	0.044*
pN1–3	42	28	
Distant metastasis:			
M0	107	111	0.122
M1	3	0	
Stage:			
I–II	86	104	0.001*
III–IV	24	7	
Histological grades	2 (1.98 ± 0.73)	2 (1.69 ± 0.70)	0.003*
Ki-67 labeling index	33.4 (36.0 ± 23.4)	19.10 (26.2 ± 20.9)	< 0.001*
Lymphatic infiltration:			
Absent	45	65	< 0.001*
Present	65	46	
Venous infiltration:			
Absent	78	104	< 0.001*
Present	32	7	
ER:			
Negative	18	17	0.543
Positive	92	94	
PgR:			
Negative	33	34	0.313
Positive	77	77	
HER2:			
Negative	91	98	0.294
Positive	17	12	

^a^ Median (mean ± SD),

* *p* < 0.05

## Discussion

Aromatase is a member of the cytochrome p450 family of enzymes, and it is the key enzyme that converts androgens to estrogens in the breast cancer environment [21, 22]. Geisler et al. reported that the intratumor estradiol level of ER-positive breast cancer samples was correlated with tumor aromatase expression, as evaluated by their own anti-aromatase antibody 677 [16]. Aromatase inhibitors are now the most important hormonal drugs used to manage postmenopausal patients with luminal breast cancer [23]. However, hormone-sensitive cases are selected clinically based on assessments of the ER and PgR statuses of the resected tissue instead of aromatase status. Attempts to generate clinically applicable monoclonal antibodies to aromatase have been unsatisfactory [9, 14, 15]. Here, we prepared clinically applicable polyclonal antisera on an industrial production scale.

Osawa and colleagues prepared a catalytically suppressive anti-human placental aromatase monoclonal antibody (MAb3-2C2) [10] and, using immunoaffinity chromatography, isolated aromatase from human placental microsomes to a homogeneity greater than 97% with a 300- to 400-fold increase in specific activity [11]. Polyclonal antisera raised by immunizing rabbits with the purified aromatase showed high affinity and specificity for human aromatase [12] and have been used for immunohistochemistry in various normal and neoplastic tissues [12, 13]. In our project to produce new antisera, we followed the main procedures for preparing the reference R-8-1 antibody that had been demonstrated to be potent and specific against human aromatase [11–13]. We used a Sepharose 4B column coupled with MAb3-2C2, a monoclonal antibody specific to human aromatase [10]. One of the minor modifications was that SDS was added to purified aromatase fractions to a final concentration of 0.01% to avoid adsorption of aromatase to containers such as plastic tubes at the final step of purification, in which the purified aromatase was likely to be denatured. Thus, each of the antisera from Rabbits 1, 2, and 3 was raised using SDS-denatured aromatase as an immunogen. In contrast, the reference antiserum R-8-1 was raised using active (intact) aromatase as an immunogen. As a result, the reactivity of each of the antisera from Rabbits 1, 2, and 3 was slightly different from that of antiserum R-8-1. Thus, the affinities of each of the antisera from Rabbits 1, 2, and 3 to purified aromatase shown by ELISA were the same as, or better than that of antiserum R-8-1. In addition, aromatase from the placental microsome extract was likely to be active; consequently, the affinities of the rabbit antisera to placental microsome extract as shown by ELISA were considerably lower than the antiserum R-8-1. The affinity of Rabbit 1, 2 and 3 antisera to SDS-denatured aromatase was higher than that of R-8-1. Most of the aromatase in formalin-fixed paraffin embedded (FFPE) tissue is denatured form. Rabbit 3 is the best candidate for aromatase immunohistochemistry of FFPE material.

In the breast cancer microenvironment, both cancerous and stromal cells, such as fat cells and fibroblasts, are known to produce aromatase [24, 25]. However, which cell type is more important for breast cancer progression is controversial [24]. Our immunohistochemistry showed that aromatase was localized predominantly in the cytoplasm of neoplastic cells. Epithelial aromatase expression was consistently more intense than in the stromal tissues. These findings are consistent with immunohistochemical studies using antiserum R-8-2 in various human tissues including the placenta [20], ovary [26], uterine endometrium [12], endometriotic tissue [12], adenomyosis [12], and ovarian epithelial carcinoma [27]. Furthermore, our present findings indicate that normal breast epithelium and neoplastic cells are the major source of aromatase in breast cancer tissues and that intracellular estrogen production probably stimulates cancer growth in a paracrine or intracrine fashion.

We demonstrated immunoreactivity to aromatase in 87.3% of the breast cancer cases, although the immunostaining intensity varied; this was more frequent than in previous estimates. No significant correlation was found between aromatase-positive cases and ER- or PgR-positive cases. Our results showed that intense aromatase expression was associated with greater age, lower histological grade, lower Ki-67 index, lower lymphatic infiltration rate, and lower venous infiltration rate. Aromatase expression might represent tumor differentiation independent from ER and/or PgR status.

The majority of aromatase studies have been performed with cultured cells or animal models. Clinical studies of aromatase expression have been limited and the significance of aromatase expression levels for explaining clinicopathological factors is controversial. Esteban et al. studied 38 breast cancers immunohistochemically using their own polyclonal antibody [9]. They reported an inverse correlation between intratumoral aromatase expression and ER status, but no significant correlation was detected with regard to age, tumor size, lymph node status, stage, or PgR status. We did not detect a statistically significant association between aromatase and ER or PgR levels. Some studies have reported correlations between age and aromatase expression of breast cancers similar to our data [28–30], but others did not [9, 31]. These inconsistencies might be caused by the different antibodies and detection systems used in the various studies [24]. The localization of aromatase has been controversial. Miki et al. reported that aromatase mRNA could be detected both in cancer parenchyma and in stromal cells including cancer associated fibroblasts [25]. The localization of aromatase varies among cases by immunohistochemistry using their antibody 677. The aromatase expression in breast cancer parenchyma was almost constantly greater than stromal cells in our present study. These differences in results were probably caused by the different antibodies used. Our antibody was raised using aromatase denatured by SDS treatment and might be sensitive for degenerated aromatase but not for native form. Aromatase immunohistochemistry of FFPE material might depend on the aromatase degeneration status caused by formalin fixation or heating during tissue processing.

This study has two limitations. We used tissue microarrays for aromatase immunohistochemistry. A previous report using antibody 677 concluded that tissue microarrays were not suitable for assaying *in situ* aromatase expression [17]. In the present study, we used a novel antiserum, and the expression rate and staining pattern were different from the studies using antibody 677. However, we need to interpret the results cautiously. Another potential problem was in the specificity of the antiserum. We have not yet tested this antiserum for other ER-positive tumors such as ovarian or endometrial cancers, so further studies on such tumors are desirable.

In conclusion, we have generated a clinically applicable polyclonal antiserum to aromatase with high affinity and specificity. In breast cancer specimens, 87.3% of the cases had positive immunoreactivity for aromatase. Intense aromatase immunoreactivity was associated with better clinicopathological factors but it was independent from ER and PgR statuses. Immunohistochemistry of aromatase will be a useful clinical marker in addition to ER and PgR to select various treatment options. Larger-scale studies will be necessary for assessing the significance of aromatase expression in patients with breast cancers.

## Supporting information

S1 Fig N-terminal amino acid sequence of aromatase. Purified aromatase was separated by SDS–PAGE and electrotransferred to a PVDF membrane. The band flanking c. 50 kDa was subjected to N-terminal amino acid sequencing and showed two patterns of amino acid sequences, Val·Leu·Glu·Met·Leu and Met·Leu·Asn·Pro·Ile, consistent with the n-1 and n-4 forms of aromatase, respectively.
(TIF)

S1 Table Aromatase expression and clinicopathological features of the cases with invasive breast cancers. (XLSX)
