# Correction: Preparation of a novel antiserum to aromatase with high affinity and specificity: Its clinicopathological significance on breast cancer tissue

**DOI:** 10.1371/journal.pone.0183202

**Published:** 2017-08-08

**Authors:** Naoki Kanomata, Shiro Matsuura, Tsunehisa Nomura, Junichi Kurebayashi, Taisuke Mori, Jo Kitawaki, Takuya Moriya

The abstract appears incorrectly. The correct Abstract is: Aromatase inhibitors have been widely used for the endocrine treatment of estrogen-dependent breast cancer in postmenopausal patients. However, clinicopathological studies of aromatase have been limited due to unsatisfactory specificity and/or restricted availability of anti-aromatase antibodies. Here, we have generated a polyclonal antiserum with high affinity and specificity for human aromatase using a monoclonal antibody tagged immunoaffinity chromatography on an industrial production scale. Our preliminary immunohistochemical analysis of 221 invasive breast cancer cases indicated that 87.3% (193/221) had at least 5% aromatase positive cells. The histoscore for aromatase was inversely correlated with pT (*p* = 0.019), pN (*p* = 0.001), stage (*p* < 0.001), histologic grade (*p* = 0.003), lymphatic infiltration (*p* < 0.001), venous infiltration (*p* < 0.001), and Ki-67 index (*p* < 0.001). However, cancer aromatase expression was independent of estrogen receptor (ER), progesterone receptor (PgR), and human epidermal growth factor receptor 2 statuses. This antiserum will be applicable to clinicopathological examination of aromatase in addition to ER and PgR for an appropriate use of aromatase inhibitor on the treatment of breast cancer. Further studies on the relationship between aromatase expression and aromatase inhibitors are warranted.
