# Supplementary figures and images for: Preparation of a novel antiserum to aromatase with high affinity and specificity: Its clinicopathological significance on breast cancer tissue
Source: PLoS One. 2017 May 10;12(5):e0177439. doi: 10.1371/journal.pone.0177439 (PMC5425223; doi:10.1371/journal.pone.0177439)

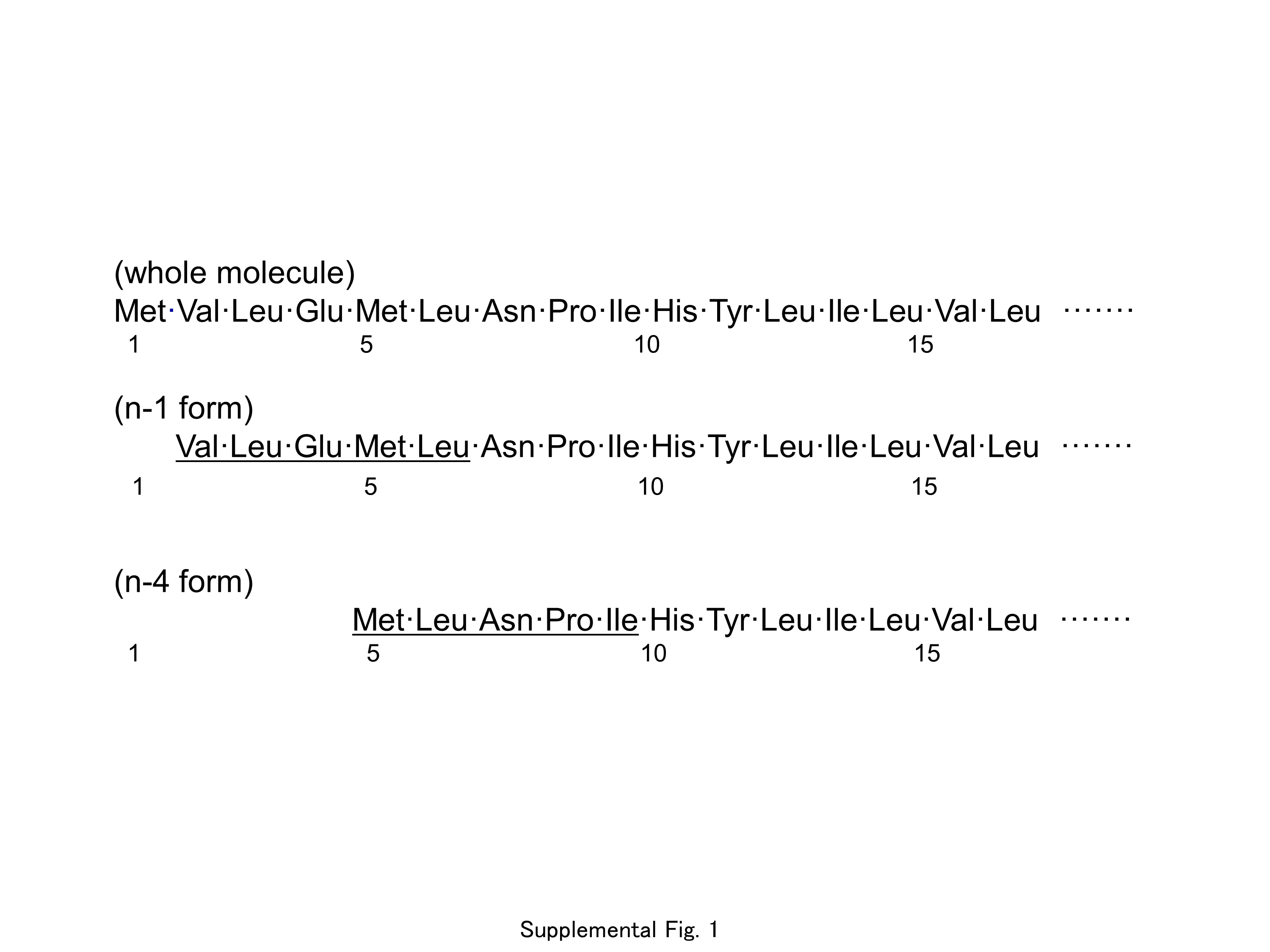

Supplement: S1 Fig — Purified aromatase was separated by SDS–PAGE and electrotransferred to a PVDF membrane. The band flanking c. 50 kDa was subjected to N-terminal amino acid sequencing and showed two patterns of amino acid sequences, Val·Leu·Glu·Met·Leu and Met·Leu·Asn·Pro·Ile, consistent with the n-1 and n-4 forms of aromatase, respectively. (TIF) [file pone.0177439.s001.TIF]
